# 3-Hy­droxy-*N*′-[(*E*)-3-pyridyl­methyl­idene]-2-naphtho­hydrazide

**DOI:** 10.1107/S1600536811025591

**Published:** 2011-07-06

**Authors:** Chuan Li, Xiuyun Zhang, Qingkun Wu, Handong Yin

**Affiliations:** a College of Chemistry and Chemical Engineering, Liaocheng University, Shandong 252059, People’s Republic of China

## Abstract

The title compound, C_17_H_13_N_3_O_2_, displays an *E* configuration about the C=N bond. The mean planes of the pyridine and benzene rings make a dihedral angle of 31.2 (2)°. An intra­molecular O—H⋯O hydrogen bond is observed. In the crystal, inter­molecular N—H⋯N hydrogen bonding links the mol­ecules into a chain along [101].

## Related literature

For related structures, see: Lv *et al.* (2006 ▶); Tarafder *et al.* (2002 ▶); Zhou *et al.* (2009 ▶); Huang (2009 ▶); Shafiq *et al.* (2009 ▶); Liang *et al.* (2008 ▶).
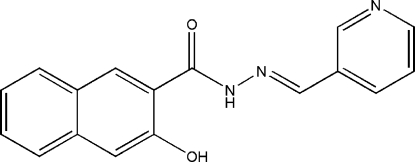


## Experimental

### 

#### Crystal data

C_17_H_13_N_3_O_2_
*M* *_r_* = 291.30
Orthorhombic, 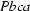

*a* = 11.0976 (11) Å
*b* = 10.4422 (9) Å
*c* = 23.9903 (17) Å
*V* = 2780.1 (4) Å^3^
*Z* = 8
Mo *K*α radiation
μ = 0.09 mm^−1^
*T* = 298 K
0.39 × 0.38 × 0.32 mm

#### Data collection

Bruker SMART CCD area-detector diffractometer
Absorption correction: multi-scan (*SADABS*; Sheldrick, 1996 ▶) *T*_min_ = 0.964, *T*_max_ = 0.971
10663 measured reflections
2451 independent reflections
1486 reflections with *I* > 2σ(*I*)
*R*_int_ = 0.043

#### Refinement

*R*[*F* ^2^ > 2σ(*F* ^2^)] = 0.039
*wR*(*F* ^2^) = 0.109
*S* = 1.07
2451 reflections
200 parameters
H-atom parameters constrained
Δρ_max_ = 0.16 e Å^−3^
Δρ_min_ = −0.18 e Å^−3^

### 

Data collection: *SMART* (Bruker, 2007 ▶); cell refinement: *SAINT* (Bruker, 2007 ▶); data reduction: *SAINT*; program(s) used to solve structure: *SHELXS97* (Sheldrick, 2008 ▶); program(s) used to refine structure: *SHELXL97* (Sheldrick, 2008 ▶); molecular graphics: *SHELXTL* (Sheldrick, 2008 ▶); software used to prepare material for publication: *SHELXTL*.

## Supplementary Material

Crystal structure: contains datablock(s) I, global. DOI: 10.1107/S1600536811025591/kp2338sup1.cif

Structure factors: contains datablock(s) I. DOI: 10.1107/S1600536811025591/kp2338Isup2.hkl

Supplementary material file. DOI: 10.1107/S1600536811025591/kp2338Isup3.cml

Additional supplementary materials:  crystallographic information; 3D view; checkCIF report

## Figures and Tables

**Table 1:** Hydrogen-bond geometry (Å, °)

*D*—H⋯*A*	*D*—H	H⋯*A*	*D*⋯*A*	*D*—H⋯*A*
O2—H2⋯O1	0.82	1.87	2.6015 (18)	147
N1—H1⋯N3^i^	0.86	2.12	2.956 (2)	165

Symmetry code: (i) 
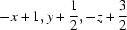
.

**Fig. 1.**
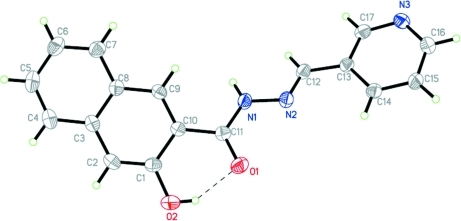
The molecular structure of the title compound with 50% probability displacement ellipsoids. An O—H···O intramolecular hydrogen bond is shown as a dashed line.

**Fig. 2.**
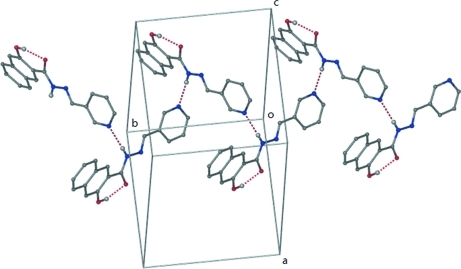
View of the chain formed by N—H···N hydrogen bond.

## supplementary crystallographic information

### Comment

Schiff bases have been extensively investigated because of their important applications in coordination chemistry, catalysis and biological processes. (Lv *et al.*, 2006; Tarafder *et al.*, 2002; Zhou *et al.*, 2009). In continuation of our research of organotin derivatives, the title compound, a novel Schiff base, was prepared and its crystal structure presented (Fig. 1). The structure of the molecule shows an *E* configuration about the C=N bond. The mean planes of the pyridine and benzene rings make the dihedral angle of 31.20 (15)°. Intramolecular O—H···O hydrogen bond was observed (Fig. 1 and Table 1). All bond lengths and angles are normal and correspond to those observed in the related componds (Huang (2009); Shafiq *et al.*, 2009; Liang *et al.*, 2008). In the crystal structure, intermolecular N—H···N hydrogen bonds link the molecules into a chain (Fig.2 and Table 1).

### Experimental

The methanol solution of 3-pyridinecarboxaldehyde(0.2 mol) was added dropwise to a solution of 3-hydroxy-2-naphthohydrazide(0.2 mol) in mehtanol. Then the mixture was stirred at room temperature for 6 h, during which time a brown precipitate was observed. The precipitate was filtrated off and the obtained solid was recrystallised from methanol. Anal. Calc (%) for C_17_H_13_N_3_O_2_ (291.30): C, 70.11; H, 4.45; N, 14.48. Found (%): C, 70.09; H, 4.50; N, 14.43.

### Refinement

All data were corrected using *SADABS* method and the final refinement was performed by mull-matrix least-square method with anisotropic parameters for non-hydrogen atoms on F2 using *SHELX97* program. The hydrogen atoms were added theoretically, riding on the concerned atoms and refined with fixed thermal factors (*U*_iso_(H) = 1.2 *U*_eq_(C, N); *U*_iso_(H) = 1.5 *U*_eq_(O)).

### Crystal data

**Table:**

C_17_H_13_N_3_O_2_	*F*(000) = 1216
*M**_r_* = 291.30	*D*_x_ = 1.392 Mg m^−^^3^
Orthorhombic, *P**b**c**a*	Mo *K*α radiation, λ = 0.71073 Å
Hall symbol: -P 2ac 2ab	Cell parameters from 2315 reflections
*a* = 11.0976 (11) Å	θ = 2.5–24.7°
*b* = 10.4422 (9) Å	µ = 0.09 mm^−^^1^
*c* = 23.9903 (17) Å	*T* = 298 K
*V* = 2780.1 (4) Å^3^	Block, brown
*Z* = 8	0.39 × 0.38 × 0.32 mm

### Data collection

**Table:**

Bruker SMART CCD area-detector diffractometer	2451 independent reflections
Radiation source: fine-focus sealed tube	1486 reflections with *I* > 2σ(*I*)
graphite	*R*_int_ = 0.043
phi and ω scans	θ_max_ = 25.0°, θ_min_ = 2.5°
Absorption correction: multi-scan (*SADABS*; Sheldrick, 1996)	*h* = −13→13
*T*_min_ = 0.964, *T*_max_ = 0.971	*k* = −12→12
10663 measured reflections	*l* = −16→28

### Refinement

**Table:**

Refinement on *F*^2^	Primary atom site location: structure-invariant direct methods
Least-squares matrix: full	Secondary atom site location: difference Fourier map
*R*[*F*^2^ > 2σ(*F*^2^)] = 0.039	Hydrogen site location: inferred from neighbouring sites
*wR*(*F*^2^) = 0.109	H-atom parameters constrained
*S* = 1.07	*w* = 1/[σ^2^(*F*_o_^2^) + (0.033*P*)^2^ + 1.2301*P*] where *P* = (*F*_o_^2^ + 2*F*_c_^2^)/3
2451 reflections	(Δ/σ)_max_ = 0.018
200 parameters	Δρ_max_ = 0.16 e Å^−^^3^
0 restraints	Δρ_min_ = −0.18 e Å^−^^3^

### Fractional atomic coordinates and isotropic or equivalent isotropic displacement parameters (Å^2^)

**Table:**

	*x*	*y*	*z*	*U*_iso_*/*U*_eq_	
N1	0.72696 (12)	0.13202 (13)	0.68223 (5)	0.0454 (4)	
H1	0.6567	0.1573	0.6719	0.055*	
N2	0.73782 (12)	0.03490 (13)	0.72100 (5)	0.0441 (4)	
N3	0.51884 (13)	−0.26353 (14)	0.83141 (6)	0.0517 (4)	
O1	0.92858 (10)	0.16623 (12)	0.67932 (5)	0.0587 (4)	
O2	0.99947 (10)	0.37468 (13)	0.62980 (5)	0.0631 (4)	
H2	1.0011	0.3162	0.6526	0.095*	
C1	0.89726 (14)	0.36595 (16)	0.59886 (7)	0.0436 (5)	
C2	0.88220 (15)	0.44499 (16)	0.55447 (7)	0.0478 (5)	
H2A	0.9415	0.5053	0.5466	0.057*	
C3	0.77958 (15)	0.43855 (15)	0.51990 (6)	0.0425 (5)	
C4	0.76205 (18)	0.51752 (17)	0.47290 (7)	0.0559 (5)	
H4	0.8195	0.5790	0.4640	0.067*	
C5	0.66274 (19)	0.50506 (18)	0.44061 (7)	0.0611 (6)	
H5	0.6528	0.5587	0.4100	0.073*	
C6	0.57495 (18)	0.41305 (17)	0.45245 (7)	0.0594 (6)	
H6	0.5079	0.4048	0.4295	0.071*	
C7	0.58781 (16)	0.33592 (16)	0.49747 (7)	0.0513 (5)	
H7	0.5288	0.2754	0.5054	0.062*	
C8	0.68986 (15)	0.34632 (15)	0.53254 (6)	0.0409 (4)	
C9	0.70638 (15)	0.26731 (15)	0.57943 (6)	0.0415 (5)	
H9	0.6471	0.2076	0.5881	0.050*	
C10	0.80619 (14)	0.27488 (15)	0.61284 (6)	0.0378 (4)	
C11	0.82639 (15)	0.18724 (16)	0.66058 (6)	0.0420 (5)	
C12	0.64006 (16)	−0.00150 (16)	0.74330 (6)	0.0447 (5)	
H12	0.5686	0.0406	0.7347	0.054*	
C13	0.63850 (14)	−0.10870 (15)	0.78229 (6)	0.0388 (4)	
C14	0.74226 (16)	−0.16753 (17)	0.80160 (7)	0.0478 (5)	
H14	0.8176	−0.1357	0.7918	0.057*	
C16	0.62105 (17)	−0.31857 (17)	0.84855 (7)	0.0539 (5)	
H15	0.6158	−0.3916	0.8706	0.065*	
C17	0.53001 (16)	−0.15897 (18)	0.79985 (7)	0.0486 (5)	
H17	0.4599	−0.1172	0.7890	0.058*	
C15	0.73317 (16)	−0.27280 (17)	0.83522 (7)	0.0525 (5)	
H18	0.8020	−0.3128	0.8489	0.063*	

### Atomic displacement parameters (Å^2^)

**Table:**

	*U*^11^	*U*^22^	*U*^33^	*U*^12^	*U*^13^	*U*^23^
N1	0.0402 (8)	0.0517 (8)	0.0444 (7)	0.0054 (7)	0.0001 (6)	0.0108 (7)
N2	0.0446 (8)	0.0480 (8)	0.0397 (7)	0.0038 (7)	0.0002 (7)	0.0052 (7)
N3	0.0461 (8)	0.0557 (9)	0.0533 (8)	−0.0090 (8)	0.0024 (7)	0.0052 (8)
O1	0.0406 (7)	0.0751 (8)	0.0603 (7)	0.0044 (7)	−0.0062 (6)	0.0105 (7)
O2	0.0445 (7)	0.0711 (8)	0.0737 (8)	−0.0115 (7)	−0.0106 (6)	0.0042 (7)
C1	0.0365 (9)	0.0458 (9)	0.0486 (10)	0.0007 (8)	0.0019 (8)	−0.0083 (8)
C2	0.0469 (10)	0.0396 (9)	0.0569 (10)	−0.0067 (9)	0.0099 (9)	−0.0016 (9)
C3	0.0494 (10)	0.0368 (9)	0.0414 (9)	0.0028 (8)	0.0086 (8)	−0.0026 (8)
C4	0.0716 (13)	0.0470 (10)	0.0490 (10)	0.0013 (10)	0.0134 (10)	0.0065 (9)
C5	0.0886 (14)	0.0533 (11)	0.0415 (10)	0.0139 (11)	0.0013 (10)	0.0065 (9)
C6	0.0766 (13)	0.0561 (11)	0.0455 (10)	0.0086 (11)	−0.0126 (10)	−0.0063 (9)
C7	0.0564 (11)	0.0456 (10)	0.0518 (10)	0.0003 (9)	−0.0067 (9)	−0.0038 (9)
C8	0.0472 (10)	0.0384 (9)	0.0372 (8)	0.0035 (8)	0.0021 (8)	−0.0045 (8)
C9	0.0426 (10)	0.0385 (9)	0.0434 (9)	−0.0058 (8)	0.0038 (8)	−0.0025 (8)
C10	0.0366 (9)	0.0372 (9)	0.0394 (8)	0.0006 (8)	0.0036 (7)	−0.0029 (7)
C11	0.0389 (9)	0.0458 (10)	0.0412 (9)	0.0008 (8)	0.0014 (8)	−0.0041 (8)
C12	0.0416 (10)	0.0499 (10)	0.0427 (9)	0.0049 (9)	−0.0043 (8)	0.0042 (8)
C13	0.0376 (9)	0.0444 (9)	0.0344 (8)	0.0011 (8)	−0.0011 (7)	−0.0007 (8)
C14	0.0373 (9)	0.0569 (11)	0.0491 (9)	−0.0016 (9)	0.0003 (8)	0.0072 (9)
C16	0.0593 (12)	0.0473 (10)	0.0552 (11)	−0.0049 (10)	−0.0015 (9)	0.0075 (9)
C17	0.0395 (10)	0.0602 (11)	0.0462 (9)	0.0004 (9)	−0.0042 (8)	−0.0008 (9)
C15	0.0427 (10)	0.0543 (11)	0.0606 (11)	0.0043 (9)	−0.0052 (9)	0.0130 (9)

### Geometric parameters (Å, °)

**Table:**

N1—C11	1.349 (2)	C6—C7	1.355 (2)
N1—N2	1.3814 (17)	C6—H6	0.9300
N1—H1	0.8600	C7—C8	1.415 (2)
N2—C12	1.268 (2)	C7—H7	0.9300
N3—C17	1.334 (2)	C8—C9	1.407 (2)
N3—C16	1.336 (2)	C9—C10	1.370 (2)
O1—C11	1.2395 (19)	C9—H9	0.9300
O2—C1	1.3586 (19)	C10—C11	1.483 (2)
O2—H2	0.8200	C12—C13	1.459 (2)
C1—C2	1.358 (2)	C12—H12	0.9300
C1—C10	1.428 (2)	C13—C17	1.379 (2)
C2—C3	1.411 (2)	C13—C14	1.385 (2)
C2—H2A	0.9300	C14—C15	1.367 (2)
C3—C4	1.410 (2)	C14—H14	0.9300
C3—C8	1.418 (2)	C16—C15	1.371 (2)
C4—C5	1.353 (3)	C16—H15	0.9300
C4—H4	0.9300	C17—H17	0.9300
C5—C6	1.397 (3)	C15—H18	0.9300
C5—H5	0.9300		
			
C11—N1—N2	120.11 (13)	C7—C8—C3	119.14 (15)
C11—N1—H1	119.9	C10—C9—C8	122.64 (15)
N2—N1—H1	119.9	C10—C9—H9	118.7
C12—N2—N1	115.43 (14)	C8—C9—H9	118.7
C17—N3—C16	116.59 (15)	C9—C10—C1	118.26 (14)
C1—O2—H2	109.5	C9—C10—C11	122.59 (14)
C2—C1—O2	119.38 (15)	C1—C10—C11	119.04 (14)
C2—C1—C10	120.14 (15)	O1—C11—N1	122.22 (15)
O2—C1—C10	120.48 (15)	O1—C11—C10	121.84 (15)
C1—C2—C3	122.12 (15)	N1—C11—C10	115.94 (14)
C1—C2—H2A	118.9	N2—C12—C13	120.71 (15)
C3—C2—H2A	118.9	N2—C12—H12	119.6
C4—C3—C2	123.62 (16)	C13—C12—H12	119.6
C4—C3—C8	118.11 (15)	C17—C13—C14	117.05 (15)
C2—C3—C8	118.26 (14)	C17—C13—C12	119.88 (15)
C5—C4—C3	120.91 (17)	C14—C13—C12	123.02 (15)
C5—C4—H4	119.5	C15—C14—C13	119.52 (16)
C3—C4—H4	119.5	C15—C14—H14	120.2
C4—C5—C6	121.16 (17)	C13—C14—H14	120.2
C4—C5—H5	119.4	N3—C16—C15	123.29 (17)
C6—C5—H5	119.4	N3—C16—H15	118.4
C7—C6—C5	119.82 (18)	C15—C16—H15	118.4
C7—C6—H6	120.1	N3—C17—C13	124.45 (16)
C5—C6—H6	120.1	N3—C17—H17	117.8
C6—C7—C8	120.84 (17)	C13—C17—H17	117.8
C6—C7—H7	119.6	C14—C15—C16	119.00 (17)
C8—C7—H7	119.6	C14—C15—H18	120.5
C9—C8—C7	122.31 (15)	C16—C15—H18	120.5
C9—C8—C3	118.54 (15)		
			
C11—N1—N2—C12	172.76 (15)	O2—C1—C10—C9	−178.18 (15)
O2—C1—C2—C3	178.39 (15)	C2—C1—C10—C11	178.32 (15)
C10—C1—C2—C3	−1.8 (2)	O2—C1—C10—C11	−1.9 (2)
C1—C2—C3—C4	−178.81 (16)	N2—N1—C11—O1	−9.7 (2)
C1—C2—C3—C8	0.2 (2)	N2—N1—C11—C10	170.29 (13)
C2—C3—C4—C5	178.62 (17)	C9—C10—C11—O1	156.80 (16)
C8—C3—C4—C5	−0.4 (2)	C1—C10—C11—O1	−19.4 (2)
C3—C4—C5—C6	−0.5 (3)	C9—C10—C11—N1	−23.2 (2)
C4—C5—C6—C7	1.1 (3)	C1—C10—C11—N1	160.67 (14)
C5—C6—C7—C8	−0.6 (3)	N1—N2—C12—C13	176.15 (13)
C6—C7—C8—C9	−179.64 (16)	N2—C12—C13—C17	−170.56 (16)
C6—C7—C8—C3	−0.3 (2)	N2—C12—C13—C14	6.9 (2)
C4—C3—C8—C9	−179.83 (15)	C17—C13—C14—C15	1.8 (2)
C2—C3—C8—C9	1.1 (2)	C12—C13—C14—C15	−175.73 (15)
C4—C3—C8—C7	0.8 (2)	C17—N3—C16—C15	0.0 (3)
C2—C3—C8—C7	−178.27 (15)	C16—N3—C17—C13	2.9 (2)
C7—C8—C9—C10	178.50 (16)	C14—C13—C17—N3	−3.8 (2)
C3—C8—C9—C10	−0.9 (2)	C12—C13—C17—N3	173.85 (15)
C8—C9—C10—C1	−0.7 (2)	C13—C14—C15—C16	0.7 (3)
C8—C9—C10—C11	−176.86 (15)	N3—C16—C15—C14	−1.7 (3)
C2—C1—C10—C9	2.0 (2)		

### Hydrogen-bond geometry (Å, °)

**Table:**

*D*—H···*A*	*D*—H	H···*A*	*D*···*A*	*D*—H···*A*
O2—H2···O1	0.82	1.87	2.6015 (18)	147
N1—H1···N3^i^	0.86	2.12	2.956 (2)	165

Symmetry codes: (i) −*x*+1, *y*+1/2, −*z*+3/2.
